# Cross-Breed Few-Shot Learning for Pig Detection via Improved YOLOv7 and CycleGAN-Based Sample Generation

**DOI:** 10.3390/biology15080623

**Published:** 2026-04-16

**Authors:** Yizheng Zhuang, Lingyao Xu, Jinyun Jiang, Zhenyang Zhang, Yiting Wang, Pengfei Yu, Yihan Fu, Haoqi Xu, Wei Zhao, Xiaoliang Hou, Jianlan Wang, Yongqi He, Yan Fu, Zhe Zhang, Qishan Wang, Yuchun Pan, Zhen Wang

**Affiliations:** 1 College of Animal Sciences, Zhejiang University, Hangzhou 310058, China; 22317058@zju.edu.cn (Y.Z.);; 2 Hainan Institute of Zhejiang University, Building 11, Yongyou Industrial Park, Yazhou Bay Science and Technology City, Yazhou District, Sanya 572025, China; 3 SciGene Biotechnology Co., Ltd., Hefei 230031, China

**Keywords:** pig detection, YOLOv7 optimization, few-shot learning, CycleGAN augmentation, precision farming

## Abstract

Complex farming environments, breed variation, and the high cost of manual annotation remain major obstacles to robust pig detection, while cross-breed detection under few-shot conditions has been insufficiently explored in previous studies. To address this gap, we propose a few-shot pig detection framework that combines an improved YOLOv7 detector with CycleGAN-based pseudo-sample generation. The detector was enhanced through anchor optimization, Efficient Channel Attention (ECA), and Log-Sum-Exp (LSE) pooling to improve localization and feature discrimination in dense pigsty scenes. In addition, an optimized CycleGAN with perceptual loss was used to generate synthetic Duroc-like pig images to enrich the limited target-domain training set. The framework was evaluated using a two-dataset design: a White Pig Base Dataset was used to establish the source-domain detector and validate the architectural improvements, whereas a Duroc Pig Few-Shot Dataset was used to assess cross-breed adaptation under a 10-shot setting. The experimental results show that the proposed method achieved 98.16% mAP on the White pig dataset and 85.52% mAP on the Duroc Few-Shot Dataset. On the Duroc Few-Shot Dataset, the final framework outperformed Faster R-CNN, CenterNet, and YOLOv8, and also surpassed DCGAN- and SRGAN-based augmentation strategies. These results indicate that the proposed method provides an effective and practical solution for cross-breed few-shot pig detection, with potential value for intelligent livestock monitoring under annotation-limited conditions.

## Simple Summary

Automatic pig detection is an important first step for smart livestock monitoring because it supports later tasks such as animal tracking, behavior analysis, and health observation. However, pig detection in real farms is difficult because animals often overlap, lighting conditions change, and different breeds can look very different. In addition, collecting and labeling large numbers of images for every breed is expensive and time-consuming. The aim of this study was to develop a method that can improve pig detection when only a very small number of labeled images are available for a new breed. To do this, we combined an improved detection model with a method that creates additional training images by transferring the visual appearance of one breed onto images of another breed. The results show that this approach improved pig detection performance under limited-label conditions. These findings suggest that combining model improvement with generated training images may help reduce data collection costs and support more practical video-based monitoring in pig farming.

## 1. Introduction

Pork is one of the most important animal protein sources worldwide [1], and large-scale pig farming increasingly depends on Precision Livestock Farming (PLF) technologies to improve production efficiency, management precision, and animal welfare [2]. Among these technologies, automated video-based monitoring provides a practical and non-invasive means of continuously observing livestock status under commercial farm conditions [3]. Integrating intelligent technologies in large-scale pig farming is critical for enhancing operational efficiency, improving animal welfare, and minimizing feed waste [4]. By integrating computer vision with artificial intelligence, such systems can support real-time assessment of animal behavior, health, and production-related conditions, thereby reducing labor demands and improving management responsiveness. In this context, accurate pig detection is a fundamental prerequisite for downstream tasks such as individual localization, trajectory tracking, and behavior analysis in intelligent livestock monitoring systems.

Various object detection methods, including Faster R-CNN [5], the YOLO series [6,7,8], and Mask R-CNN [9], have been applied to livestock monitoring and adapted to pig-related tasks. However, pigsty environments pose unique challenges, such as significant variations in lighting, small and indistinct pig appearances, and frequent occlusions and overlaps among animals [10]. To address these complexities, research has focused on refining object detection algorithms for practical use in livestock farming. Hansen et al. [11] utilized convolutional neural networks (CNNs), pre-trained models (e.g., VGG-Face), and data augmentation to achieve high-precision pig face recognition, though the study primarily relied on overhead surveillance video due to the difficulty of collecting face images in farming settings. Yang et al. [12] leveraged Faster R-CNN and designed an algorithm associating pig heads with bodies, enhancing individual localization in group housing using accessible overhead video footage. Further developments have explored advanced deep learning techniques and attention mechanisms. Nasirahmadi et al. [13] employed InceptionV2, ResNet, and InceptionResNet V2 for pig posture detection, enabling the identification of standing, side-lying, and prone postures from 2D imaging systems. Wei et al. [14] introduced an efficient multi-scale attention (EMA) module into the YOLOv8n model, improving detection capabilities in complex scenarios. Lu et al. [15] addressed background interference during detection by employing a rotational anchor frame model, mitigating the challenges posed by redundant data and complex environments. In addition to these environmental difficulties, inter-breed differences in coat color, texture, and local appearance patterns may further weaken the transferability of visual features learned from one breed to another. The lack of sufficient annotated data that aligns with specific environments, stages, and breeds negatively impacts model performance [16]. Therefore, the challenge is not limited to annotation scarcity alone; it also lies in the combined effect of breed-dependent visual shift and data scarcity, which remains insufficiently addressed in existing pig detection studies.

Currently, few-shot learning methods, including meta-learning [17], transfer learning [18], attention models [19], data augmentation [20], and metric learning [21], have emerged as promising approaches. For example, Han et al. [22] developed the SBeA framework, which identifies animal identities and estimates 3D postures and social behavior maps using minimal annotated frames, demonstrating the feasibility of low-annotation learning in animal-related vision tasks. In addition to these approaches, adjacent paradigms such as unsupervised domain adaptation [23] and robust learning under noisy labels [24] have further highlighted the broader importance of handling domain shift and annotation uncertainty in dense visual tasks. Unsupervised domain adaptation [25] aims to bridge the gap between labeled source domains and unlabeled target domains without requiring target-domain annotation [25], whereas noise-robust learning addresses the performance degradation caused by imperfect annotations in crowded or ambiguous scenes [26]. Recent work has emphasized the growing importance of these directions in practical visual learning systems. However, most existing pig detection studies still rely on relatively sufficient annotations, focus on single-breed or fixed-environment settings, or evaluate models under limited domain shifts. Taken together, these studies provide an important foundation, but cross-breed pig detection under extreme few-shot conditions remains insufficiently explored.

Therefore, an important gap remains in developing pig detection models that can adapt across breeds under annotation-limited conditions. In practical production settings, a detector trained on a source breed may not transfer reliably to a target breed because substantial differences in coat color, texture, and local appearance can alter the learned feature distribution. This problem is further exacerbated when only a few target-domain images are available, as the model must cope simultaneously with environmental complexity and cross-breed visual shift under severe data scarcity [27]. Under such circumstances, improving the detector alone may be insufficient, whereas relying only on a very small target-domain dataset may lead to unstable optimization and limited performance transfer [28]. A more practical strategy is therefore to jointly strengthen detector-side feature learning and enrich target-domain appearance diversity through data augmentation.

**Figure 1:**
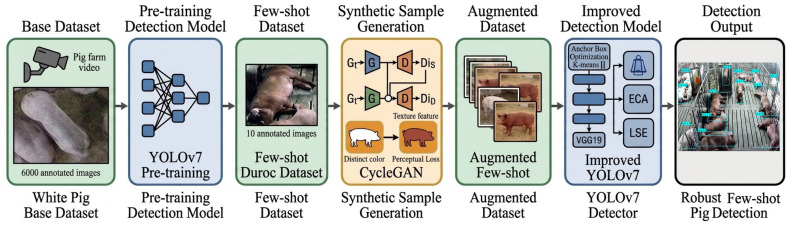
Process flowchart of the improved Pig Few-Shot learning object detection method.

To address this problem, we developed a cross-breed few-shot pig detection framework that combines detector adaptation with pseudo-sample generation. Specifically, we improved YOLOv7 through anchor optimization, Efficient Channel Attention (ECA), and Log-Sum-Exp (LSE) pooling to enhance localization and feature discrimination in dense pigsty scenes. We further employed a CycleGAN-based image translation strategy with perceptual loss to generate Duroc-like pseudo-samples from White pig images, thereby enriching the target-domain training data under a few-shot setting. A two-dataset design was adopted in which a White Pig Base Dataset was used to establish and validate the source-domain detector, whereas a Duroc Pig Few-Shot Dataset was used to evaluate cross-breed adaptation under limited annotations. Figure 1 illustrates the overall workflow of the proposed framework. Overall, this study aims to provide an effective and practical framework for cross-breed pig detection in annotation-limited livestock monitoring scenarios.

## 2. Materials and Methods

### 2.1. Materials

#### 2.1.1. Video Acquisition

**Figure 2:**
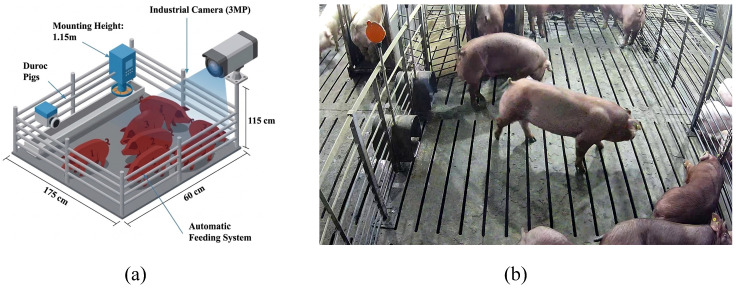
Data collection system for pig detection. (**a**) Structural layout of the pig pen showing camera placement, mounting height, and integration with the automatic feeding system. (**b**) Representative image captured in real farming conditions with multiple Duroc pigs.

As shown in Figure 2a,b, the original RGB videos of pigs under pen with a single-space automatic feeding system (space size: 175 × 60 × 115 cm) were collected using a 3-megapixel TP-LINK industrial video camera with a 4 mm focal length (TL-IPC632-A, TP-Link, Shenzhen, China). These videos depicted pigs at fattening stage between 27 November and 24 December 2023, at the nucleus farm of SciGene Biotechnology company (Hefei, China). The primary focus of video collection was on Duroc pigs, which represent the key monitoring target in actual production scenarios. The camera recorded at a frame rate of 30 frames per second and a resolution of 2304 × 1296 pixels. To minimize obstructions from the automatic feeding system and enhance coverage, it was placed diagonally from the rear of the pen during filming, approximately 1.15 m above the ground.

#### 2.1.2. Dataset Construction

**Figure 3:**
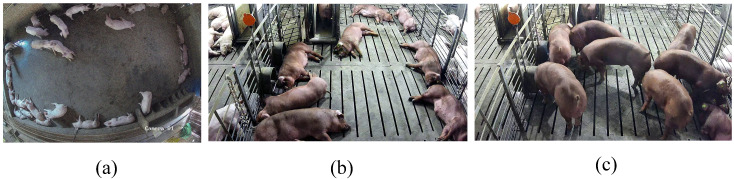
Construction of the White Pig Base Dataset and the Duroc Pig Few-Shot Dataset. (**a**) Sample White pig images from the 6040 raw collected frames. (**b**,**c**) Representative frames from the Duroc Pig Few-Shot Dataset.

Figure 3a shows examples of the raw White pig images collected from publicly accessible online resources related to pig farming, including agricultural websites, publicly available pig farming images, and video materials showing real or near-real farm environments. To improve dataset relevance, only images representing realistic pig production scenarios were retained after manual screening, whereas images with severe blur, unrealistic rendering, non-farming backgrounds, or insufficient visible pig information were excluded. A total of 6040 raw White pig images were initially collected, and 6000 high-quality images were retained after quality control and annotated using Labelme [29] according to a standardized protocol. The dataset was divided into a training set (70%, 4200 images) and a hold-out evaluation set (30%, 1800 images) using stratified random sampling to preserve the distribution of major scene characteristics, including illumination conditions, pig density, and posture-related scene complexity. This ratio ensures sufficient training data for stable feature learning and reliable evaluation of model generalization (±1.2% mAP, α = 0.05). This split was used to support stable detector training and consistent evaluation under the current source-domain setting.

For cross-breed few-shot learning, 37 frames were selected from surveillance videos to construct the Duroc Pig Few-Shot Dataset, with annotations for Duroc pigs and White pigs. The selected frames were sampled to cover representative variations in illumination, spatial position, and overlap conditions while avoiding temporally adjacent frames whenever possible to reduce redundancy among samples. In terms of illumination, the selected frames included both relatively uniform lighting conditions and scenes with partial shadows or uneven brightness caused by the farm environment. Because each image typically contained multiple pigs, individual animals within the same frame often exhibited mixed postural states, including standing, walking, lying, feeding, and partial occlusion, thereby increasing posture-related diversity at the scene level. In the Duroc Pig Few-Shot Dataset, the annotated Duroc pigs included 146 standing individuals, 261 lying individuals, and 45 prone individuals, whereas the annotated White pigs included 57 standing individuals, 186 lying individuals, and 19 prone individuals. Figure 3b,c shows that frames were selected to maximize diversity in lighting, positioning, and behaviors. Under this setting, the dataset was divided into 10 training images, 15 validation images, and 12 test images, corresponding to a 10-shot target-domain learning scenario commonly used in few-shot studies [30]. The larger validation set (15 images) allows for precise hyperparameter tuning and early stopping, mitigating overfitting risks. The 12-image test set provides a representative assessment of generalization. Although the dataset originated from a single camera and a single-pen context, frame selection was designed to capture representative within-scene variation rather than consecutive near-duplicate images. A fixed random seed was used for splitting to ensure reproducibility and avoid distribution bias. All annotations were originally in JSON format, including bounding box coordinates, object categories, and frame metadata. These were converted to YOLO-compatible TXT format (class, xmin, ymin, xmax, ymax) using a custom Python script, with correctness validated by comparing random samples before and after conversion.

### 2.2. Methods

#### 2.2.1. Improved YOLOv7

YOLOv7 was selected as the baseline model for this study due to its superior speed and accuracy on benchmark datasets [8]. It performs well on many common object detection benchmark datasets [31] and is renowned for its speed and efficiency [32], which is crucial in livestock scenarios where rapid detection of a large number of animals is required. However, the complex visual characteristics of pigsty scenes, including posture variation, dense distribution, occlusion, and breed-related appearance differences, make the default YOLOv7 configuration suboptimal for cross-breed pig detection under few-shot conditions. Therefore, three modifications were introduced to the baseline architecture: anchor box optimization, Efficient Channel Attention (ECA), and Log-Sum-Exp (LSE) pooling.

**Figure 4:**
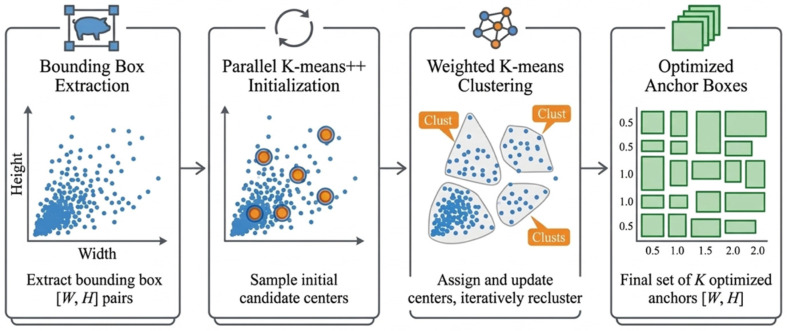
Pipeline for anchor box optimization using parallel k-means|| and weighted k-means clustering.

Figure 4 illustrates the process of re-designing anchor boxes to better accommodate the considerable variation in pig size, body shape, and posture. All bounding boxes in the training set were extracted based on their width and height, normalized to the network input resolution (640 × 640), and clustered using the k-means|| algorithm to generate an optimized set of anchors. This data-driven procedure produces anchor configurations that more accurately reflect the true distribution of pig aspect ratios—including standing, lying, lateral-recumbent, and prone postures—compared with the default COCO anchors. The optimized anchors substantially reduce shape mismatch and enhance the detector’s ability to localize elongated or irregular pig silhouettes.

**Figure 5:**
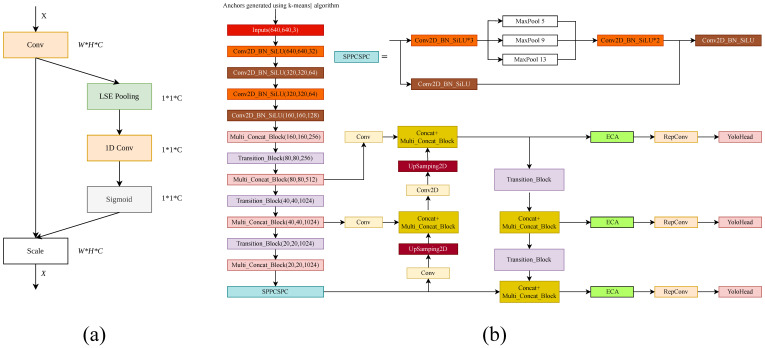
Architecture of the proposed detector. (**a**) Structure of the integrated ECA–LSE attention module. (**b**) Overall network architecture with anchor optimization, ECA modules, and modified feature-extraction pathways.

Figure 5a illustrates the integration of the Efficient Channel Attention (ECA) module into the YOLOv7 backbone to enhance its representational capacity for pig detection, particularly under challenging conditions such as variations in coat color, hair texture, and lighting. ECA is a lightweight channel-attention mechanism that avoids dimensionality reduction and computes inter-channel dependencies using a 1D convolution applied to global average-pooled features. By learning channel-wise weights in this efficient manner, ECA enables the network to selectively amplify informative feature channels while suppressing less relevant ones, thereby improving discrimination between visually similar pig breeds (e.g., Duroc versus White pigs).

In our implementation, three ECA modules were inserted after the outputs of the dark2, dark3, and dark4 backbone stages to refine channel responses at multiple levels of the feature hierarchy (Figure 5b). The corresponding feature maps were approximately 160 × 160 × 256, 80 × 80 × 512, and 40 × 40 × 1024, respectively, whereas the final dark5 stage was kept unchanged (Figure 5b). To further improve detection in dense settings—where pigs frequently overlap or occlude one another—the original global pooling operation was replaced with Log-Sum-Exp (LSE) pooling [33]. LSE pooling provides a smooth interpolation between max pooling (which highlights dominant spatial activations) and average pooling (which aggregates global context), enabling the network to better capture both salient features and contextual cues from closely clustered individuals.

Because LSE pooling changes the tensor form used for channel weighting, auxiliary tensor operations such as transpose and unsqueeze were introduced to maintain compatibility with the subsequent one-dimensional convolution and downstream layers. The combined ECA–LSE design improves the balance between local discriminative information and global contextual cues, thereby supporting more stable feature extraction in crowded pig detection scenarios.

#### 2.2.2. Few-Shot Dataset Augmentation via CycleGAN and Loss Optimization

The study involved two pig breeds: White pigs, which are abundant in the base dataset, and Duroc pigs, which are underrepresented due to field constraints and high annotation costs. Although both breeds share similar body structures, Duroc pigs exhibit darker, coarse, and curly hair with distinctive pigmentation, whereas White pigs have uniform light-colored coats. These visual differences limit the generalization of features learned from White pigs when applied to Duroc pigs, particularly under few-shot conditions. Traditional data augmentation techniques are insufficient to generate the necessary diversity, motivating the use of generative adversarial networks (GANs) to synthesize additional training samples [34].

**Figure A1:**
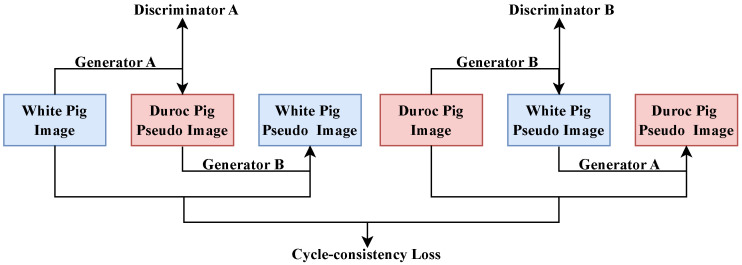
Schematic illustration of the CycleGAN framework used for pseudo-sample generation between white pig and Duroc pig images.

To address this challenge, we employed CycleGAN [35], a cyclic adversarial network capable of unsupervised image-to-image translation without paired training samples (Figure A1). CycleGAN training was performed in an unpaired setting, where domain A corresponded to White pig images and domain B corresponded to Duroc pig images. Images from the two domains were sampled independently from domain-specific image lists without one-to-one correspondence. All training images were converted to RGB, resized to 512 × 512 pixels using bicubic interpolation, and then used for cross-domain translation learning. To improve convergence under the limited target-domain setting, the generators and discriminators were initialized from pretrained horse-to-zebra weights. Training was conducted for 200 epochs with a batch size of 1 using the Adam optimizer, with an initial learning rate of 2 × 10^−4^, a minimum learning rate of 2 × 10^−6^, cosine learning-rate decay, β1 = 0.5, β2 = 0.999, and weight decay set to 0.

**Figure 6:**
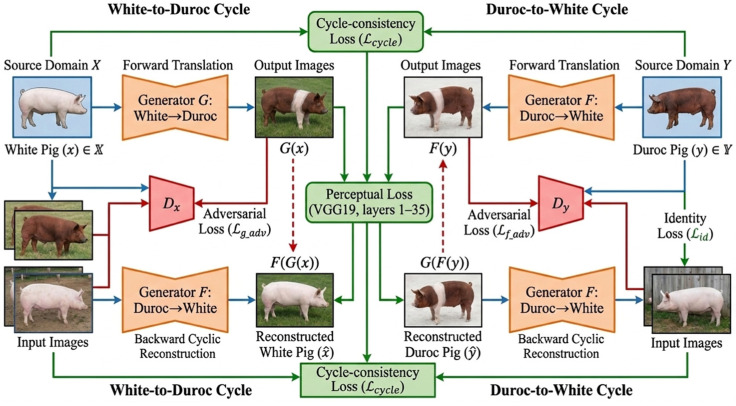
Architecture of the improved CycleGAN framework with perceptual loss for cross-breed pig image translation and pseudo-sample generation.

In addition to the adversarial, identity, and cycle-consistency objectives, a perceptual loss term was introduced for the A-to-B translation branch to improve the perceptual quality of generated pseudo-samples. This perceptual loss was implemented using a pretrained VGG19 network, from which the first 35 feature layers were extracted and frozen (Figure 6) [36]. The perceptual term was computed as the mean squared error between the VGG19 feature representations of the generated image and the target-domain reference image. The final generator objective combined identity loss, adversarial loss, cycle-consistency loss, and perceptual loss. Specifically, identity losses for the two domains were weighted by 5.0, adversarial losses for the A-to-B and B-to-A branches were weighted by 1.0 and 0.5, respectively, cycle-consistency losses for the two directions were each weighted by 10.0, and the perceptual loss for the A-to-B branch was weighted by 0.5. The first 35 VGG19 feature layers were adopted in order to retain relatively rich structural and texture-related information in feature space while avoiding reliance on only very shallow or highly abstract layers.

After CycleGAN training, pseudo-Duroc images were generated using the trained A-to-B generator, which translated selected White pig source images into Duroc-like images. During inference, each source image was resized to 512 × 512 pixels, processed by the generator, and then restored to its original spatial resolution. Because this style-transfer process primarily modified appearance attributes such as coat color and texture while preserving the overall image geometry and object layout, the bounding box annotations of the corresponding White pig source images were directly inherited for the generated pseudo-samples. In total, 50 pseudo-samples were added to the few-shot training set to increase target-domain appearance diversity and support subsequent YOLOv7 training.

#### 2.2.3. Evaluation Metrics for Object Detection and Pseudo-Sample Quality

The evaluation of pig object detection in intensive farming environments differs from standard real-time detection scenarios due to the relatively slow movement of pigs and the complex backgrounds, including overlapping animals, occlusions, and grid floors. Consequently, this study prioritizes detection accuracy over processing speed, as most modern hardware can achieve sufficient frame rates for farm monitoring.

Detection performance was quantified using Intersection over Union (IoU) and mean Average Precision (mAP). IoU measures the overlap between a predicted bounding box and its corresponding ground truth and is defined as

(1) $$IoU=\frac{Area\;of\;Overlap}{Area\;of\;Union}$$

A higher IoU indicates more precise localization of individual pigs. Multiple IoU thresholds were used to determine true positive detections, providing a robust assessment of localization accuracy in complex pigsty environments.

Average Precision (AP) summarizes the precision–recall relationship for each class:

(2) $$AP_{i}=\int_{0}^{1}P_{i}(R)dR$$

where $P_{i}(R)$ represents the precision at recall R for class i. The mean Average Precision (mAP) across all N classes is then calculated as

(3) $$mAP=\frac{1}{N}\sum_{i=1}^{n}APi$$

For this study, the COCO-style mAP@0.5:0.95 was adopted, averaging AP over ten IoU thresholds ranging from 0.5 to 0.95 in increments of 0.05, providing a comprehensive evaluation of detection performance for pigs of varying sizes, postures, and occlusion levels. Additional evaluations at specific thresholds, such as 0.5 and 0.75, were also reported to facilitate direct comparisons with previous studies. To ensure statistical robustness, all experiments were repeated five times with different random seeds, and the mean mAP, standard deviation, and 95% confidence intervals were calculated:

(4) $$CI_{95\%}=\bar{x}\pm 1.96\frac{\sigma }{\sqrt{n}}$$

where $\bar{x}$ is the mean mAP, σ is the standard deviation across runs, and n is the number of repetitions. These repeated runs account for variability introduced by weight initialization, data shuffling, and pseudo-sample generation. Because the Duroc Pig Few-Shot Dataset was derived from a single-camera and a single-pen context, the reported standard deviations and confidence intervals should be interpreted as repeated-run variability under the current evaluation split, rather than the full uncertainty of broader cross-environment generalization. To reduce temporal redundancy among subsets, the few-shot images were sampled with non-contiguous frame intervals whenever possible.

The evaluation was conducted for the baseline YOLOv7, the improved YOLOv7 with optimized anchor boxes, the ECA–LSE refinement modules, and the augmented dataset with synthetic pseudo-samples, allowing for a systematic assessment of each module’s contribution. Additionally, paired *t*-tests were performed to examine the significance of improvements over baseline models, with *p* < 0.01 considered statistically significant.

In addition to downstream detection accuracy, the quality of CycleGAN-generated pseudo-samples was assessed using Fréchet Inception Distance (FID) and Inception Score (IS). FID evaluates the distributional similarity between generated images and target-domain images, with lower values indicating closer alignment to the target-domain distribution. IS evaluates the diversity and discriminability of generated samples, with higher values indicating better image quality in terms of classifiable structure and variation. These metrics were used to compare the original and improved CycleGAN models and to complement the downstream detection results with a quantitative assessment of pseudo-sample quality.

All metrics were computed using the COCO evaluation framework, a standardized and widely adopted methodology for multi-class object detection in complex environments [37]. This framework, combined with multiple runs, confidence intervals, and statistical testing, ensures that reported improvements in detection accuracy are both reproducible and statistically robust.

## 3. Results

### 3.1. Experimental Platform and Experimental Configuration

The experimental setup was conducted on a laboratory server equipped with two NVIDIA A100 PCIe GPUs (40 GB memory each; NVIDIA, Santa Clara, CA, USA), interconnected via NVLink, and an Intel Xeon Platinum 8260 CPU with 48 cores (Intel, Santa Clara, CA, USA), with 512 GB of DDR4 RAM. The software environment included Ubuntu 20.04.6 LTS, CUDA v12.0, cuDNN v8.5, and PyTorch v1.10.1 on Python v3.8.18, with additional tools such as TensorBoard for monitoring training logs. To ensure reproducibility, random seeds were fixed across all experiments. Model checkpoints were saved every five epochs to facilitate recovery and evaluation. The training process employed Distributed Data Parallel (DDP) with NCCL for GPU synchronization, and mixed precision training was enabled via NVIDIA Apex to optimize memory usage and computation speed. The experimental setup is shown in Table 1.

In the few-shot Duroc pig object detection task, we leveraged a transfer learning approach to fine-tune the YOLOv7 network, taking advantage of the similarity between the White Pig Base Dataset and the Duroc Pig Few-Shot Dataset. The backbone of the YOLOv7 model was frozen during the initial training phase to maintain the stability of the pre-trained feature extraction network, while the rest of the network was fine-tuned. The training process was conducted in two stages: (1) Frozen Training: The backbone was frozen, and the model was trained for 200 epochs with a batch size of 1. This phase focused on adapting the head layers to the Duroc Pig Dataset. (2) Unfrozen Training: In the second phase, the entire network was unfrozen, and training continued for another 200 epochs with a batch size of 4. The initial learning rate in the frozen stage was set to 5 × 10^−4^, with a minimum learning rate of 5 × 10^−6^, whereas in the unfrozen stage, the initial learning rate was adjusted to 6.25 × 10^−4^, with a minimum learning rate of 6.25 × 10^−6^. A cosine learning-rate decay schedule was used throughout training. The total training length of 400 epochs was determined empirically based on preliminary convergence behavior: shorter training was insufficient for stable target-domain adaptation, whereas longer training did not consistently yield additional improvement.

Because the target-domain dataset was extremely limited, the small batch size in the frozen stage should be regarded as a practical compromise rather than an intrinsically optimal configuration. This setting may introduce noisier gradient updates, but it was adopted to preserve feasible optimization under the available few-shot data and hardware constraints. Other optimization settings, including the optimizer, input size, and data-processing pipeline, were kept consistent with the source-domain detector training unless otherwise specified. The main training configuration is summarized in Table 2.

### 3.2. Performance on the White Pig Base Dataset

The White Pig Base Dataset was first used to evaluate the effect of the architectural modifications applied to YOLOv7 under the source-domain setting. Under otherwise matched experimental conditions, the baseline YOLOv7 model achieved 96.79% mAP.

After incorporating the optimized anchor boxes (Table A1), detection accuracy increased to 97.72%, corresponding to a 0.93% improvement over the baseline. The refined anchors better matched the empirical distribution of pig bounding boxes, thereby improving localization under the current source-domain setting.

Further integration of the Efficient Channel Attention (ECA) module and LSE pooling elevated the mAP to 98.16%, representing a total performance gain of 1.37% over the original YOLOv7 model. In the ablation results, the increment introduced by ECA alone over the anchor-optimized model was small, whereas the additional inclusion of LSE pooling produced a clearer performance increase. Under the current source-domain setting, this pattern suggests that channel-wise refinement alone contributed modestly, while the ECA–LSE combination was more beneficial for feature extraction in crowded pig scenes. Table 3 summarizes the effect of these architectural modifications on YOLOv7.

To characterize repeated-run variability under the current evaluation split, all experiments were repeated five times with different random seeds. The improved model achieved an average performance of 98.16% ± 0.48% mAP (95% CI). A paired *t*-test confirmed that the performance gains introduced by the optimized architecture were statistically significant relative to the baseline (*p* < 0.01). Here, the reported confidence interval reflects repeated-run variability under the current evaluation setting rather than the full uncertainty of broader out-of-domain generalization. In addition to detection accuracy, computational efficiency was also examined. Under the experimental platform described in Section 3.1, inference speed was measured on the laboratory server equipped with two NVIDIA A100 PCIe GPUs (40 GB memory each), using an input image size of 640 × 640 pixels. Under this setting, the modified YOLOv7 model contained approximately 36.7 M parameters, achieved an inference speed of 32 FPS, and required about 8 GB of GPU memory during inference. These results indicate that the added modules introduced only a modest computational cost on the current server-grade hardware. However, because the present evaluation was conducted on high-performance GPUs, the reported 32 FPS should not be interpreted as direct evidence of deployment readiness for resource-constrained edge devices. Further model compression or acceleration strategies, such as pruning, quantization, or lightweight redesign, may be required for embedded farm-side deployment. A summary of the accuracy and efficiency changes before and after the model improvements is provided in Table 4.

**Figure 7:**
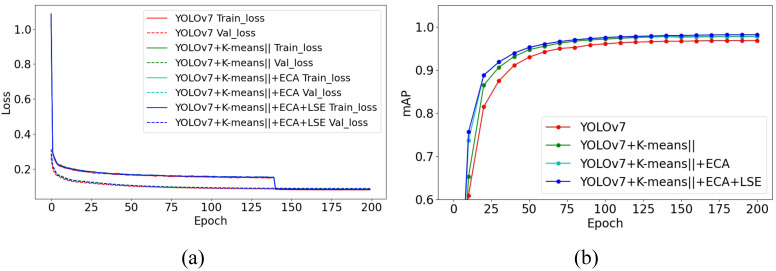
Ablation results on the White Pig Base Dataset. (**a**) Training and validation loss curves. (**b**) mAP curves for different model variants.

Figure 7 presents the ablation analysis of the proposed YOLOv7 architectural improvements on the White Pig Base Dataset. From the Loss curves, it can be observed that each modification reduces both training and validation losses, indicating faster convergence and improved learning stability.

**Figure 8:**
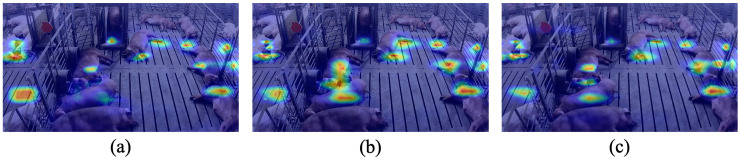
Representative feature-response heatmaps for different model variants in a crowded pig scene. (**a**) Anchor-optimized model. (**b**) Anchor + ECA. (**c**) Anchor + ECA–LSE.

To further examine the effect of the proposed feature-refinement design, representative feature-response heatmaps were visualized for three detector variants: the anchor-optimized model, the anchor-optimized model with ECA, and the anchor-optimized model with ECA–LSE refinement (Figure 8). Compared with the anchor-only model, the ECA-enhanced model showed more concentrated activation over pig body regions, especially for partially overlapping individuals in the central area of the pen. After further introducing LSE pooling, the anchor + ECA–LSE model exhibited clearer response continuity over pig bodies and reduced diffuse activation over background structures such as the slatted floor and pen boundaries. These visualizations are consistent with the quantitative ablation results and suggest that the integrated ECA–LSE design was beneficial for feature refinement in crowded pig scenes.

### 3.3. Few-Shot Detection Performance on the Duroc Dataset

The proposed architecture was further evaluated on the Duroc Pig Few-Shot Dataset, which contained 10 training images, 15 validation images, and 12 test images extracted from surveillance video streams. To reduce temporal redundancy across subsets, frames were sampled from separated temporal segments while avoiding temporally adjacent images whenever possible. Because the dataset originated from a single camera and a single pen context, the following results should be interpreted as within-scenario performance under the current few-shot setting rather than as evidence of broad cross-environment generalization.

Using the baseline YOLOv7 model, the overall detection accuracy on the Few-Shot Dataset reached 60.18% mAP. After applying anchor box optimization, detection accuracy for Duroc pigs (B-pig) increased markedly from 0.57 to 0.89, demonstrating that the optimized anchors more accurately captured the geometric characteristics of Duroc pig body shapes. However, performance for White pigs (W-pig) decreased from 0.64 to 0.52, indicating that anchor optimization alone may introduce a bias toward the dominant morphology when training data are extremely limited.

**Figure 9:**
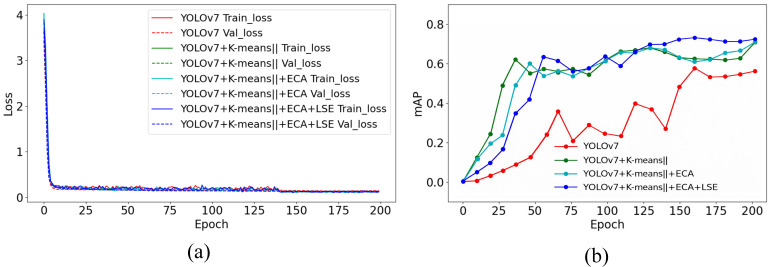
Ablation results on the Duroc Pig Few-Shot Dataset. (**a**) Training and validation loss curves. (**b**) mAP curves for different model variants.

After further introducing the ECA–LSE refinement modules, W-pig detection accuracy recovered to 0.64, while B-pig performance remained high at 0.84. Under the current few-shot Duroc setting, this pattern suggests that the combined refinement modules improved the balance of feature extraction across pigs with different visual appearances. Consequently, the overall detection accuracy increased to 74.31% mAP, representing a 14.13% improvement over the baseline YOLOv7 model (Table 5 and Table 6, Figure 9).

To characterize repeated-run variability under the same dataset partition, all experiments were repeated five times using different random seeds. The enhanced model achieved an average accuracy of 74.31% ± 0.8% mAP (95% confidence interval). A paired *t*-test confirmed that the improvements over the baseline were statistically significant (*p* < 0.01). Despite the extremely limited training data, the improved YOLOv7 architecture exhibited stable and consistent detection performance. Here, the reported standard deviation and confidence interval reflect repeated-run variability under the same dataset partition rather than the full uncertainty of broader cross-farm or cross-environment generalization. Therefore, these results support the effectiveness of the proposed modifications within the current few-shot Duroc evaluation scenario. Compared with the source-domain evaluation, the stronger benefit observed in the target-domain few-shot setting suggests that the integrated refinement design was particularly helpful when appearance shift and annotation scarcity occurred simultaneously.

### 3.4. Effect of CycleGAN-Based Pseudo-Sample Generation

To further improve detection accuracy under extremely limited training data, synthetic pseudo-samples were generated using the proposed CycleGAN-based style transfer framework, which transfers the visual characteristics of Duroc pigs onto White pig images. Without any synthetic augmentation, the improved YOLOv7 model achieved a detection accuracy of 74.31% mAP on the Few-Shot Dataset. After incorporating pseudo-samples generated using the original CycleGAN model, accuracy increased to 79.68% mAP, with AP scores of 0.69 for White pigs and 0.91 for Duroc pigs. Under the current few-shot setting, this result suggests that pseudo-sample augmentation enriched the target-domain appearance distribution and helped the detector better capture breed-related visual cues.

**Figure 10:**
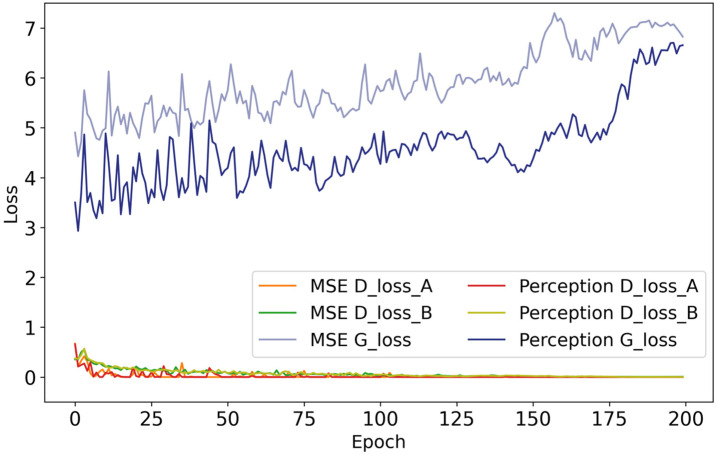
CycleGAN loss function before and after improvement. (A) MSE Loss. (B) Perception loss.

**Figure 11:**
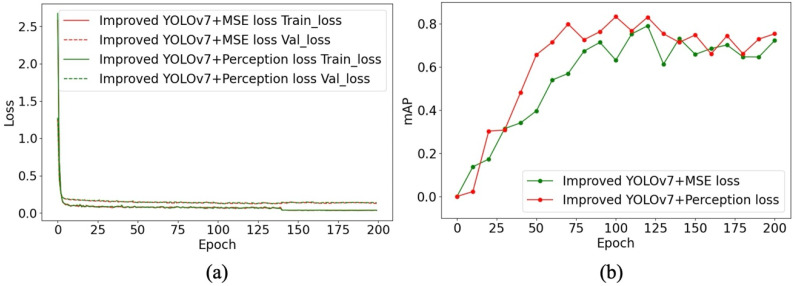
Training results on the Duroc Pig Few-Shot Dataset after pseudo-sample augmentation. (**a**) Training and validation loss curves. (**b**) mAP curves.

To enhance the visual fidelity of the generated images, the CycleGAN loss function was further refined by replacing the original pixel-level Mean Squared Error (MSE) loss with a perceptual loss computed from the first 35 layers of a pre-trained VGG19 network (Figure 10). With this modification, overall detection accuracy improved to 85.52% mAP, with AP scores of 0.79 for White pigs and 0.92 for Duroc pigs (Table 7). Relative to the baseline few-shot detector without pseudo-sample augmentation, this corresponds to an 11.21% improvement, indicating that the revised loss design was beneficial for downstream detector training in the current low-data scenario (Figure 11).

To complement the visual comparison, pseudo-sample quality was further quantified using Fréchet Inception Distance (FID) and Inception Score (IS). The original CycleGAN achieved an FID of 17.02 and an IS of 2.13 ± 0.22, whereas the improved CycleGAN achieved an FID of 15.90 and an IS of 2.28 ± 0.28. The lower FID and higher IS of the improved model indicate that the generated pseudo-samples were more consistent with the target-domain image distribution while also showing improved diversity and discriminability. These quantitative results (Table 8) are consistent with the downstream detection improvement observed after augmentation.

To investigate the effect of synthetic dataset size, an ablation study was conducted by generating 10, 30, and 50 pseudo-samples using the improved CycleGAN model. The resulting detection accuracies were 83.49%, 85.0%, and 85.52% mAP, respectively. These results (Table 9) indicate that increasing the quantity of synthetic images leads to performance gains, although improvements begin to plateau beyond approximately 30 generated samples. This suggests that a moderate number of high-quality pseudo-samples is sufficient to improve model generalization in few-shot settings.

To characterize repeated-run variability under the same dataset partition, all experiments were repeated five times using different random seeds. The CycleGAN-augmented model achieved an average performance of 85.52% ± 0.7% mAP (95% confidence interval). Here, the reported confidence interval reflects repeated-run variability under the current evaluation split rather than the full uncertainty of broader cross-environment generalization.

**Figure 12:**
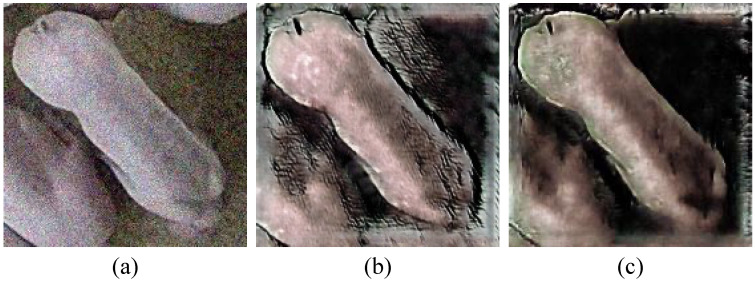
Representative pseudo-Duroc samples before and after loss-function refinement. (**a**) White pig image. (**b**) Pseudo-Duroc image generated by the original model. (**c**) Pseudo-Duroc image generated by the improved model.

Qualitative detection results are presented in Figure 12. Compared with the original CycleGAN output, the improved model produced pseudo-Duroc images with more coherent transfer of coat color and texture patterns, which is consistent with the corresponding FID and IS improvement.

**Figure 13:**
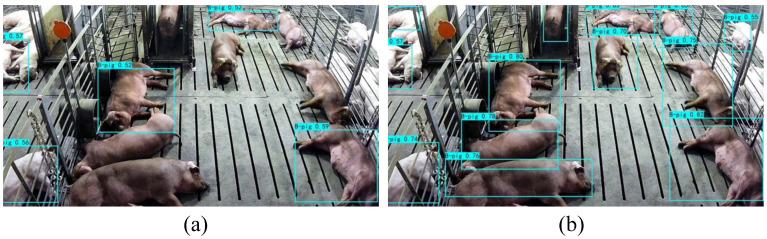
Detection results before and after applying the full framework. (**a**) Baseline model. (**b**) Improved framework.

Figure 13 compares the detection performance before and after applying the proposed improvements. In the baseline YOLOv7 results (Figure 13a), multiple pigs were missed or partially occluded, especially in densely populated pen areas. After integrating anchor optimization, channel attention, LSE pooling, and high-quality synthetic pseudo-samples (Figure 13b), the network produces more precise bounding boxes, reduces false positives, and effectively distinguishes overlapping pigs, demonstrating the overall effectiveness of the improved detection framework.

### 3.5. Comparative Experiments

To comprehensively evaluate the proposed framework, comparative experiments were conducted from two perspectives: (1) object detection algorithms and (2) pseudo-sample generation strategies. All models were trained under a matched evaluation protocol as far as permitted by their implementation characteristics, including the same dataset split, training duration, optimizer family, and data augmentation strategy. This design was used to improve comparability across models under the current experimental setting.

#### 3.5.1. Comparison with Object Detection Algorithms

Three widely used object detectors—CenterNet, YOLOv8, and Faster R-CNN—were compared with the proposed improved YOLOv7 framework under the same dataset partition and evaluation procedure.

On the White Pig Base Dataset, where source-domain training data were relatively sufficient, all models achieved high performance. YOLOv8 reached 97.9% mAP, followed by Faster R-CNN (97.4%) and CenterNet (97.0%), whereas the proposed improved YOLOv7 achieved 98.16% mAP. Under the current source-domain evaluation setting, these results indicate that the proposed model performed competitively relative to the tested detectors.

In contrast, on the Duroc Few-Shot Dataset, accuracy dropped considerably for most models due to the extremely limited training data. YOLOv8 achieved only 31.45% mAP, while CenterNet and Faster R-CNN achieved 70.01% and 80.40% mAP, respectively. The proposed framework reached 85.52% mAP—outperforming Faster R-CNN by 5.12% and YOLOv8 by over 54%. Under the current few-shot target-domain evaluation protocol, these results indicate that the proposed framework provided the strongest overall performance among the tested detectors. This advantage is consistent with the combined effects of detector-side refinement and pseudo-sample augmentation evaluated in the preceding sections.

All experiments were repeated five times using different random seeds, yielding 85.52% ± 0.7% mAP (95% CI). A paired *t*-test confirmed that performance improvements over Faster R-CNN and CenterNet were statistically significant (*p* < 0.01).

Model efficiency was also evaluated under the same hardware and input setting described in Section 3.1, namely a laboratory server equipped with NVIDIA A100 PCIe GPUs and an input image size of 640 × 640 pixels. Under this evaluation setting, the proposed YOLOv7 model contained approximately 36.7M parameters and achieved an inference speed of 32.0 FPS. Under the same setting, Faster R-CNN reached 12.8 FPS and CenterNet 25.4 FPS. These results suggest that the proposed method provides a favorable balance between detection accuracy and computational efficiency on the current server-grade hardware. The detailed comparative results of the detection algorithms are presented in Table 10.

#### 3.5.2. Comparison of Pseudo-Sample Generation Methods

To assess the effect of different pseudo-sample generation strategies on few-shot detection, three generative models—DCGAN, SRGAN, and CycleGAN—were compared (Table 11). Pseudo-samples generated by DCGAN resulted in only 43.70% mAP, indicating insufficient semantic consistency and visual realism. SRGAN-generated images improved performance to 80.16% mAP, suggesting that higher-resolution synthesis provides more informative training signals.

The proposed CycleGAN-based method with perceptual loss achieved the highest performance at 85.52% mAP, outperforming SRGAN by 5.36% and DCGAN by more than 40%. Under the current experimental protocol, these results indicate that the proposed CycleGAN-based strategy provided the most effective pseudo-sample augmentation among the tested generative models for the Duroc few-shot detector. Repeated experiments across five runs yielded 85.52% ± 0.7% mAP, with improvements over DCGAN and SRGAN being statistically significant (*p* < 0.01).

## 4. Discussion

### 4.1. Performance of the Improved YOLOv7 Detector

This study shows that adapting the YOLOv7 architecture to pig-specific detection tasks improved detection performance under the evaluated farm settings. Under the current experimental design, the proposed framework achieved 85.52% mAP on the Duroc Few-Shot Dataset and 98.16% mAP on the White Pig Base Dataset. These results suggest that the proposed method is effective for improving detection performance under annotation-limited conditions, which are common in agricultural monitoring.

One important contributor to the observed performance gain is anchor box optimization, which better aligns anchor shapes with the geometric characteristics of pigs across varying postures and sizes. In practical pig-farming scenes, pigs frequently appear in diverse states, including standing, lying, feeding, and partial overlap, all of which complicate stable localization. Under the current source- and target-domain evaluations, the optimized anchor configuration appears to reduce shape mismatch between predefined anchors and pig bounding boxes, thereby improving localization performance. These findings support the value of dataset-adaptive anchor design in agricultural vision tasks.

Furthermore, integrating the ECA–LSE refinement modules was associated with additional performance improvement beyond anchor optimization alone. In the present implementation, ECA was used to recalibrate channel responses, whereas LSE pooling replaced the original global average pooling step within the modified ECA block to better balance salient activations and broader contextual information. This design was particularly relevant to dense pig scenes in which overlap and occlusion are common. Across the evaluated datasets, the ECA–LSE combination contributed to further accuracy gains, suggesting that attention-based feature refinement can be beneficial for pig detection in visually crowded farm scenarios. In addition to the ablation results, representative feature-response heatmaps were examined to provide a qualitative view of the integrated ECA–LSE effect. The anchor + ECA and anchor + ECA–LSE variants showed more concentrated activation over pig body regions and clearer response separation in crowded scenes, which is consistent with the interpretation that the refinement modules improved feature focusing under overlap-heavy conditions.

### 4.2. Effectiveness of CycleGAN-Based Pseudo-Sample Generation

Another key contribution of this study is the incorporation of CycleGAN-based pseudo-sample generation to mitigate the scarcity of annotated Duroc images. By transferring Duroc-like appearance characteristics onto White pig images, the CycleGAN model generated additional target-domain-like samples without requiring paired training data, thereby expanding appearance diversity at no additional annotation cost.

Replacing the original pixel-level loss with a perceptual loss derived from a pretrained VGG19 network was associated with further improvement in downstream detection performance after augmentation. In addition to the detector results, quantitative image-quality evaluation showed that the improved CycleGAN achieved a lower FID and a higher IS than the original CycleGAN, indicating that the generated pseudo-samples were more consistent with the target-domain distribution while also showing improved diversity and discriminability. The performance gain increased as the number of pseudo-samples increased, although the benefit began to plateau beyond approximately 30 generated samples. Under the current Duroc few-shot setting, these findings suggest that a moderate number of pseudo-samples was sufficient to provide most of the augmentation benefit. This observation is relevant for agricultural applications in which data collection and annotation are costly and labor-intensive.

Despite the observed improvements, some failure cases remained in densely crowded scenes with severe overlap, partial body visibility, and strong illumination variation. In such cases, the detector occasionally produced incomplete bounding boxes, missed closely adjacent pigs, or showed reduced separation between overlapping individuals. These observations suggest that pseudo-sample augmentation improved overall performance under the current few-shot setting, but did not fully resolve the ambiguity caused by dense spatial arrangement, occlusion, and weak local appearance cues.

### 4.3. Comparison with Existing Object Detection Approaches

Compared with the tested object detection models, the proposed framework achieved the strongest overall performance under the current Duroc few-shot evaluation protocol. While detectors such as YOLOv8, CenterNet, and Faster R-CNN performed well when source-domain training data were relatively sufficient, their performance decreased under the target-domain few-shot setting. In contrast, the proposed method retained higher detection performance through the combined use of detector-side refinement and pseudo-sample augmentation.

These results indicate that the proposed framework benefited from integrating detector-side adaptation with target-domain appearance augmentation under annotation-limited conditions. Such a strategy is relevant to agricultural computer vision, where annotated datasets are expensive and time-consuming to collect. Nevertheless, the current efficiency evaluation was conducted on server-grade hardware, and dedicated deployment-oriented optimization will be necessary before the framework can be transferred to resource-constrained edge devices in practical farm environments. Future work could extend the approach to additional pig breeds or livestock species, incorporate temporal behavior analysis, and explore more deployment-oriented lightweight optimization.

## 5. Conclusions

This study presents a few-shot pig detection framework that integrates an improved YOLOv7 detector with CycleGAN-based pseudo-sample generation to address the challenge of limited annotated data in livestock farming environments. Two datasets—a White Pig Base Dataset and a Duroc Pig Few-Shot Dataset—were constructed from surveillance imagery captured under group housing conditions. The improved YOLOv7 model incorporated optimized anchor boxes and ECA–LSE feature refinement to better accommodate pig-specific characteristics such as body shape, coat appearance, and visual texture patterns.

The experimental results show that the proposed method achieved 98.16% mAP on the White Pig Base Dataset and 85.52% mAP on the Duroc Pig Few-Shot Dataset under the current evaluation protocol. The optimized anchor configuration improved localization performance across varying pig poses, whereas ECA–LSE feature refinement contributed additional gains in crowded and visually complex pig scenes. To address the scarcity of Duroc annotations, a CycleGAN-based style transfer strategy was introduced to generate pseudo-samples by transferring Duroc-like visual attributes onto White pig images. The introduction of perceptual loss was associated with improved pseudo-sample quality, as reflected by lower FID and higher IS values, and with further improvement in downstream few-shot detection performance.

Overall, the results indicate that combining detector-side refinement with pseudo-sample augmentation can improve pig detection performance under annotation-limited conditions. This framework reduces reliance on large labeled target-domain datasets within the evaluated setting and may provide practical value for Precision Livestock Farming applications. Nevertheless, the current study was conducted under a limited target-domain scenario, and broader validation across additional pig breeds, farm environments, and camera layouts will be required before wider deployment can be claimed. Future work will extend the framework to additional breeds and multi-view settings and explore integration with behavior analysis modules for more comprehensive on-farm monitoring.

## 6. Patents

A cross-breed object detection method, device, and storage medium using CycleGAN-based pseudo-sample generation and YOLOv7 model adaptation. This invention has been officially granted to Zhejiang University (CN119904730B, granted on 17 October 2025).

## Figures and Tables

**Table 1:** Hardware and software configuration of the experimental platform.

Hardware Environment	Equipment	Software Environment	Version
CPU	Intel Xeon Platinum 8260 (48 cores)	Python	3.8.18
GPU	NVIDIA A100 PCIe ×2	PyTorch	1.10.1
GPU memory	40 GB per GPU	CUDA	12.0
System memory	512 GB DDR4	cuDNN	8.5
GPU interconnect	NVLink	Operating system	Ubuntu 20.04.6 LTS
Training framework	Distributed Data Parallel (DDP)	Mixed precision	NVIDIA Apex
Monitoring tool	TensorBoard	Backend communication	NCCL

**Table 2:** Training configuration of the proposed YOLOv7 framework.

Parameter	Frozen Training	Unfrozen Training
Backbone	Frozen	Unfrozen
Epochs	200	200
Batch size	1	4
Optimizer	SGD	SGD
Input size	640 × 640	640 × 640
Initial learning rate	5 × 10^−4^	6.25 × 10^−4^
Minimum learning rate	5 × 10^−6^	6.25 × 10^−6^
LR schedule	Cosine decay	Cosine decay
Random seed	Fixed	Fixed
Training strategy	Transfer learning	Fine-tuning

**Table 3:** Performance comparison of different YOLOv7 variants on the White Pig Base Dataset.

Model Variant	Anchor Optimization	ECA	LSE Pooling	mAP (%)
YOLOv7 (baseline)	×	×	×	96.79
YOLOv7 + Anchor	√	×	×	97.72
YOLOv7 + Anchor + ECA	√	√	×	97.73
YOLOv7 + Anchor + ECA + LSE	√	√	√	98.16

**Table 4:** Accuracy changes on the White Pig Base Dataset before and after model improvements.

Model	mAP (%)	Std	95% CI	FPS	Parameters (M)	GPU Memory (GB)
YOLOv7 (baseline)	96.79	0.32	±0.29	34	36.0	7.5
Improved YOLOv7	98.16	0.48	±0.42	32	36.7	8.0

**Table 5:** Accuracy changes on the Duroc Pig Dataset before and after model improvements.

Model	B-Pig mAP	W-Pig mAP	Overall mAP	Std	95% CI
YOLOv7 (baseline) YOLOv7 + Anchor	0.57	0.64	60.18	0.70	±0.67
	0.89	0.52	67.33	0.81	±0.76
YOLOv7 + Anchor + ECA	0.85	0.63	72.1	0.78	±0.75
YOLOv7 + Anchor + ECA + LSE	0.84	0.64	74.31	0.80	±0.80

**Table 6:** Precision and recall of different pig detection models on the Duroc Pig Dataset.

Model	B-Pig P	W-Pig P	B-Pig R	W-Pig R
YOLOv7 (baseline) YOLOv7 + Anchor	0.62	0.76	0.52	0.49
	0.92	0.64	0.84	0.37
YOLOv7 + Anchor + ECA	0.90	0.75	0.79	0.48
YOLOv7 + Anchor + ECA + LSE	0.89	0.73	0.80	0.49

**Table 7:** Improved YOLOv7 network training results after adding pseudo-samples.

Model	W-mAP	B-mAP	mAP(%)	Loss
Improved YOLOv7 + MSE Loss	0.69	0.91	79.68	0.036
Improved YOLOv7 + Perception loss	0.79	0.92	85.52	0.035

**Table 8:** Quantitative evaluation of pseudo-sample quality for different CycleGAN settings.

Model	FID	IS
Improved YOLOv7 + MSE Loss	17.02	2.13 ± 0.22
Improved YOLOv7 + Perception loss	15.90	2.28 ± 0.28

**Table 9:** Effect of synthetic dataset size on few-shot detection.

Number of Synthetic Samples	W-Pig AP	B-Pig AP	Overall mAP (%)
10	0.75	0.88	83.49
30	0.78	0.91	85.00
50	0.79	0.92	85.52

**Table 10:** Comparative experiments on detection algorithms.

Model	White Pig Base mAP (%)	Duroc Few-Shot mAP (%)	Std	95% CI	FPS	Parameters (M)
CenterNet	97.0	70.01	0.65	±0.62	25.4	35.5
Faster R-CNN	97.4	80.40	0.70	±0.68	12.8	42.2
YOLOv8	97.9	31.45	0.50	±0.48	28.1	37.0
Improved YOLOv7	98.16	85.52	0.70	±0.70	32.0	36.7

**Table 11:** Comparative experiments on pseudo-sample generation algorithms.

Model	Duroc Few-Shot mAP (%)	Std	95% CI
DCGAN	43.70	0.60	±0.58
SRGAN	80.16	0.65	±0.63
Improved CycleGAN	85.52	0.70	±0.70

## Data Availability

The data presented in this study are available on request from the corresponding author.

## Abbreviations

The following abbreviations are used in this manuscript:

ECA	Efficient Channel Attention
LSE	Log-Sum-Exp pooling
mAP	Mean Average Precision
AP	Average Precision
FPS	Frames Per Second
DDP	Distributed Data Parallel
NCCL	NVIDIA Collective Communications Library
SGD	Stochastic Gradient Descent
W-pig	White pig (breed)
B-pig	Duroc pig (breed)
MSE	Mean Squared Error
CI	Confidence Interval

## Appendix A

**Table A1:** Anchor box dimensions generated by K-means || for YOLOv7.

Anchor ID	Width (pixels)	Height (pixels)
1	22.67	69.33
2	46.27	49.19
3	30.31	99.58
4	42.64	85.83
5	85.33	56.47
6	65.67	78.33
7	45.83	132.15
8	98.67	82.92
9	72.67	125.86
